# Polyphenolic Profile and Antioxidant Activity of Whole Grape Juices from *Vitis labrusca* and Brazilian Hybrid Grapes in Two Training Systems

**DOI:** 10.3390/antiox13091132

**Published:** 2024-09-19

**Authors:** Francisco José Domingues Neto, Adilson Pimentel Junior, Cristine Vanz Borges, João Domingos Rodrigues, Ricardo Figueira, Mara Fernandes Moura, Igor Otavio Minatel, Aline Nunes, Giuseppina Pace Pereira Lima, Marco Antonio Tecchio

**Affiliations:** 1 School of Agricultural Sciences, Sao Paulo State University (UNESP), Botucatu 18610-034, SP, Brazil; fjdominguesneto@hotmail.com (F.J.D.N.); adilson.pimentel@unifio.edu.br (A.P.J.); ricardo.figueira@unesp.br (R.F.); marco.a.tecchio@unesp.br (M.A.T.); 2 School of Agriculture Sciences, Alto Vale do Rio do Peixe University (UNIARP), Caçador 89500-199, SC, Brazil; cristine.vanz@uniarp.edu.br; 3 Institute of Biosciences, Sao Paulo State University (UNESP), Botucatu 18618-970, SP, Brazil; joao.domingos@unesp.br (J.D.R.); igorminatel@hotmail.com (I.O.M.); alinenunes_bio@hotmail.com (A.N.); 4 Agronomic Institute of Campinas (IAC), Jundiaí 13214-820, SP, Brazil; mouram@iac.sp.gov.br

**Keywords:** phenolic compounds, anthocyanins, genotypes, beverages

## Abstract

The phenolic profile and antioxidant activity of whole grape juices from *Vitis labrusca* and Brazilian hybrids in two training systems were analyzed. Genotypes of *V. labrusca* (‘Bordô’ and ‘Isabel’) and Brazilian hybrids (‘IAC 138-22 Máximo’ and ‘BRS Violeta’) were grafted onto the rootstock ‘IAC 766 Campinas’ (106-8 ‘Mgt’ × *Vitis caribaea*) and trained on low and high trellis. After harvest, the grapes were destemmed and the berries macerated in a roller crusher. Following hot extraction without pressurization of the pomace and gentle pressing of the blend (skins, must, and seeds), the juices were bottled in amber glass bottles and pasteurized. The physicochemical and colorimetric parameters of the juices, as well as the levels of flavonoids, phenolic compounds, total monomeric anthocyanins, antioxidant activity, and polyphenolic profile, were evaluated. The juices were also subjected to sensory analysis (CAAE: 65549817.7.0000.5411). There was broad variation in all assessed characteristics. The results obtained demonstrate that the training system and grape genotype used in juice production are highly related to the presence of sugars, acidity, and bioactive compounds. Juices made from ‘Bordô’, ‘IAC 138-22 Máximo’ and ‘BRS Violeta’ grapes stood out from ‘Isabel’ juices, the main grape variety used in Brazilian juice and wine production. All juices contain bioactive compounds in considerable concentrations, indicating beverages with high antioxidant activity and, consequently, high biological potential, with the use of high trellis in vine cultivation potentially increasing concentrations.

## 1. Introduction

The global consumption of grape juice has been increasing in recent years, with Brazil and the United States standing out in this scenario due to their large-scale production of high-quality juices. Part of this growth is related to the numerous health benefits of these products, attributed to their phenolic compound content, substances described as having antioxidant, cardioprotective, anticancer, anti-inflammatory, and antimicrobial effects [1]. Of the grapes produced in Brazil, 51.4% are destined for processing and 48.6% for fresh consumption. In 2022, the production of grapes intended for processing (juices, wines, and derivatives) was 830.92 million kilograms, representing 57.07% of the national production. In 2023, 665 million liters of wine were processed, with a focus on American and hybrid grape varieties cultivated in 85% of vineyards [2].

In Brazil, grape juice is produced from American grapes (*Vitis labrusca*) and hybrids obtained through genetic improvement aimed at adaptation to local climate and soil [3]. Given the increasing demand for products with a high antioxidant capacity, research efforts are justified in seeking alternatives to diversify grape production. However, the significant diversity among cultivars results in grapes with different characteristics, both in flavor and coloration, which may be associated with the content and profile of polyphenolics. The grape cultivars of *V. labrusca*, ‘Isabel’ and ‘Bordô’, are widely cultivated in Brazil. To diversify products and offer more options to grape growers, the Agronomic Institute of Campinas (IAC) and the Brazilian Agricultural Research Corporation (EMBRAPA), along with their respective genetic improvement programs, have developed hybrid cultivars for wine and juice production [4,5]. Examples include ‘IAC 138-22 Máximo’ (‘Seibel 11342’ × ‘Syrah’) and ‘BRS Violeta’ (‘BRS Rúbea’ × ‘IAC 1398-21’), cultivars with high productivity and tolerance to major fungal diseases. However, there is a lack of information about phenolic compounds and antioxidant activity in juices derived from these grapes. Several studies show that phenolic compounds present in grapes can vary due to specific factors such as species, cultivar, edaphoclimatic conditions, geographic region, and vineyard management practices [6]. On the other hand, studies evaluating the influence of training systems on the content of phenolic compounds and antioxidant activity in grape juices are scarce.

The vine training system and canopy management are integral components of vineyard management, as they influence the microclimate of the canopy and, respectively, contribute to the efficient control of pests and diseases on vines. Interaction with solar radiation is directly determined by the vine canopy structure and is related to crop productivity and quality. The way in which the canopy is positioned and constructed determines the distribution of leaf surfaces exposed to sunlight, affecting light interception, carbon assimilation and photosynthesis [7], and, consequently, the biosynthesis and accumulation of phenolic compounds.

Grapes are among the fruits that stand out as a source of phenolic compounds with important biological characteristics, notably their antioxidant properties [8,9]. The phenolic composition and properties of grapes, as well as their antioxidant activity, especially those destined for juice and wine production, have been constantly studied, with increasing reports of higher quantities of phenolic compounds acting as antioxidants in grapes and their derivatives [6]. The profile of these compounds present in grapes, juices, and wines plays an important role in sensory characteristics and is responsible for some of the organoleptic properties such as color, aroma, flavor, bitterness, and astringency, which can be altered by the vine training system employed. Therefore, this study aimed to characterize grape juice regarding their physicochemical and biochemical contents, evaluate the effect of two training systems on physicochemical and biochemical properties in grape juice, and characterize the grape juices sensorially.

## 2. Materials and Methods

### 2.1. Characterization of the Experimental Area

Our experiment was conducted at the Fruit Center of the Agronomic Institute (IAC), located in the municipality of Jundiaí—São Paulo (23°06′ S, 46°55′ W, 745 m altitude). According to the Köppen classification, the climate is Cwa, with an average annual rainfall of 1400 mm, an average annual temperature of 19.5 °C, and relative humidity of 70.6%. The soil in the experimental area is classified as Dystrophic Red Latosol [10].

The vines in the experimental area were grafted onto the rootstock ‘IAC 766 Campinas’, planted with a spacing of 2.5 m between rows and 1 m between plants. The experimental design used was randomized complete blocks, in a 4 × 2 factorial scheme, corresponding to 4 scion varieties (*V. labrusca*: ‘Bordô’ and ‘Isabel’ and Brazilian hybrids: ‘IAC 138-22 Máximo’ and ‘BRS Violeta’) and two training systems, namely low cordon (trellis with three wire strands positioned at 1, 1.3, and 1.6 m above ground) and high cordon (trellis with four wire strands positioned at 1, 1.3, 1.6, and 2 m above ground), with 5 replications, each consisting of 3 plants per experimental plot.

### 2.2. Elaboration of Whole Grape Juices

For each experimental plot, 10 kg of destemmed grapes were used. After harvesting, the grapes were transported and sorted. Then, they were destemmed and crushed using a roller crusher, followed by hot extraction without pressure of the pomace for 1 h at a temperature of 60 ± 5 °C. After gently pressing the mixture (skins, must, and seeds) in a manual press, the juices were bottled while still hot in 200 mL amber glass bottles, then pasteurized (3 min at 80 °C). The bottles were sealed with pry-off aluminum caps, cooled, labeled, and the juices were stored at 5 °C until analysis (up to 30 days).

### 2.3. Colorimetric and Physicochemical Analysis

The color of the juices was determined using two methods: colorimetry and spectrophotometry. Colorimetry provided the variables L* (lightness), C* (chroma), and h° (hue) using a Minolta CR-10^®^ colorimeter (Konica, Minolta, Tokyo, Japan), while spectrophotometry on undiluted material provided optical density (OD) using a BEL Photonics^®^ SP 2000 UV/vis spectrophotometer (Thermo Fisher Scientific, Waltham, MA, USA), with 1.0 mm cuvettes. OD was measured at 420 nm (yellow), 520 nm (red), and 620 nm (violet). Color intensity (CI) was determined by summing the optical density of each sample at 420, 520, and 620 nm. Hue angle (H°) was obtained by the ratio of absorbances at 420 nm and 520 nm [11].

The physicochemical characteristics determined were the soluble solid content, identified by means of direct refractometry using a digital Atago^®^ refractometer (Schmidt Haensch, Berlin, Germany) with automatic temperature compensation, expressed in °Brix; titratable acidity identified by means of titration, with 1 mL of grape juice diluted in 100 mL of distilled water, titrated with a standardized 0.1 N sodium hydroxide (Sigma-Aldrich, St. Louis, MO, USA) solution using phenolphthalein (Sigma-Aldrich, St. Louis, MO, USA) as an indicator until the color change endpoint; and pH, identified using a Micronal B-274 pH meter (Mettler Toledo, Barueri, Sao Paulo, Brazil). These analyses were conducted following the methodology of the Adolfo Lutz Institute [12]. Reducing sugars were determined using the colorimetric Somogyi–Nelson method based on a glucose analytical curve, with readings taken at 510 nm [13] and results expressed as a percentage.

### 2.4. Analysis by UV-Visible Spectrophotometry and Profile of Phenolic Compounds

#### 2.4.1. Total Contents of Phenolic Compounds and Monomeric Anthocyanins

The total phenolic compounds content of grape juices was determined following the method of [14] using filtered juice. Absorbance was measured at 765 nm using a spectrophotometer (BEL Photonics^®^, SP 2000 UV/vis) and expressed in milligrams of gallic acid (Sigma-Aldrich, St. Louis, MO, USA) equivalent (GAE) per liter of juice (mg L^−1^), based on a gallic acid calibration curve (1.54 to 38.46 mg L^−1^). Total monomeric anthocyanins were determined using the pH-differential method and expressed as cyanidin-3-glucoside (Sigma-Aldrich, St. Louis, MO, USA) equivalents in mg L^−1^ of grape juice [15].

#### 2.4.2. Antioxidant Activity by UV-Visible Spectrophotometry

The antioxidant activity of the juices was determined using the methods described by [16] (DPPH), [17] (FRAP), and [18] (ABTS), both by Sigma-Aldrich, St. Louis, MO, USA. Initially, the juices were filtered, diluted in ultrapure water, vortexed for 1 min, incubated for 15 min in an ultrasonic bath (Fisher Scientific, Hampton, New Hampshire, USA) at 5 °C, and then centrifuged at 2000 rpm for 10 min.

For the DPPH method, a calibration curve was prepared with Trolox at 5, 10, 15, 20, and 25 μg, and the results were expressed in μg Trolox equivalent antioxidant capacity (TEAC) per mL of juice. Absorbance was measured at 517 nm and converted into the percentage of DPPH reduction using the following equation:

% DPPH reduction = (Abscontrol − Abssample)/Abscontrol × 100

The results obtained from antioxidant activity using the FRAP method were expressed as mM Fe L^−1^ of juice, and for ABTS, the Trolox analytical standard was used to construct calibration curves, with results expressed in μg TEAC per mL of juice.

#### 2.4.3. Phenolics Profile by Reverse-Phase High-Performance Liquid Chromatography (HPLC)

The separation, identification, and quantification of phenolic compounds in whole grape juices were conducted using high-performance liquid chromatography (HPLC) (Thermo Fisher Scientific, Bremen, Germany) according to [19], with modifications. Juice samples were filtered through PTFE membrane filters (0.45 μm, Millipore, Burlington, MA, USA) and injected (20 μL) into a UPLC system (Ultimate 3000 BioRS, Dionex-Thermo Fisher Scientific Inc., Waltham, MA, USA) equipped with a diode array detector (DAD) and a Luna^®^ 2.5 μm C18 (2) HST 2.0 × 50 mm column (Phenomenex^®^, Torrance, CA, USA) maintained at 39 °C. Substance elution was achieved by means of a gradient with a flow rate of 0.6 mL/min, with readings at wavelengths of 280 nm, 320 nm, 360 nm, and 520 nm. The mobile phase consisted of 0.85% phosphoric acid solution (solvent A) and 100% acetonitrile (solvent B). The gradient used for separation was 0–2.5 min: 4% B; 2.5–7.5 min: 8% B; 7.5–15 min: 12% B; 15–18 min: 15% B; 18–20 min: 20% B; 20–21 min: 25% B; 21–22 min: 35% B; 22–24 min: 65% B; 24–25 min: 65% B; 25–25.5 min: 35% B; 25.5–26 min: 0% B; and 26–27 min: 0% B. Compound identification was performed by comparing their retention times and ultraviolet spectra with commercial standards: gallic acid, 3-hydroxytyrosol, catechin, trans-cinnamic acid, caffeic acid, chlorogenic acid, p-coumaric acid, trans-ferulic acid, rutin, cyanidin-3,5-diglucoside, delphinidin-3-O-glucoside, cyanidin-3-O-glucoside, malvidin-3,5-diglucoside, peonidin-3-O-glucoside, and malvidin-3-O-glucoside, with purity ≥ 95% (Sigma-Aldrich, St. Louis, MO, USA). Substance quantification was performed using calibration curves prepared with commercial standards, and the results were expressed in mg L^−1^ of juice.

### 2.5. Sensory Analysis of Whole Grape Juices

The sensory analysis involved 70 untrained evaluators, representing a consumer population. The research was approved by the Research Ethics Committee of UNESP (CAAE: 65549817.7.0000.5411). The attributes evaluated included color, aroma, taste, body (structure), and overall acceptance of the juice samples, using a seven-point hedonic scale, with the endpoints labeled as disliked extremely (1) to liked extremely (7) [20]. Additionally, to assess the profile of each sensory evaluator, a questionnaire was administered to collect information on age range, gender, smoking status, occupation, education level, preference, and frequency of fruit juice consumption, as well as the intention to purchase each evaluated juice, with the options ranging from certainly would buy to certainly would not buy.

The samples were served at 6 ± 2 °C in transparent plastic cups, with approximately 30 mL per sample. Each evaluator received eight randomly ordered samples coded with three-digit random numbers. Drinkable water at room temperature and plain water biscuits were also provided for mouth cleaning before and between the evaluations of grape juice samples.

### 2.6. Statistical Analysis

The data were subjected to analysis of variance, and means were compared by Tukey’s test at a 5% probability level using the Sisvar computational program 6.0 [21]. Principal component analysis (PCA) was performed to assess the clustering of juice responses and to characterize interactions between scion varieties and training systems, using the Statistical Analysis Software (SAS) 4.0. Sensory analysis data of whole grape juices (hedonic scale) were compared using the Kruskal–Wallis ranks test (non-parametric test, analyzing univariate data by ranking the scores assigned to the juices). Significance was considered when *p* < 0.05. The intention-to-purchase data of the juices were analyzed by means of single linkage hierarchical clustering to assess similarity between the juices. The software used for these analyses was MiniTab 16.

## 3. Results and Discussions

### 3.1. Colorimetric, Physicochemical, and Biochemical Analyses via UV-Visible Spectroscopy in Whole Grape Juices

There was a significant interaction between scion varieties and training systems affecting soluble solids, pH, titratable acidity, reducing sugars, color (420, 520, and 620 nm), color intensity, hue, and antioxidant activity via FRAP (Table 1). Higher levels of soluble solids were observed in ‘Isabel’ and ‘BRS Violeta’ grape juices when grown using a low trellis system. Conversely, higher levels of soluble solids were found in the juices of ‘Bordô’ and ‘IAC 138-22 Máximo’ grapes cultivated using a high trellis system (Table 1). These findings highlight that the interaction between scion genotype and training system is specific to each genotype, thus not allowing for a generalized conclusion across species or hybrids, as each genotype possesses its distinct characteristics.

However, regardless of the scion genotype or training system, all juices had soluble solid values above the minimum established by Brazilian legislation for whole grape juices (14 °Brix) [22]. The soluble solid content and titratable acidity in juices from ‘IAC 138-22 Máximo’ and ‘BRS Violeta’ grapes were similar to those reported for juices produced from hybrid grapes, with a range of 10 to 21.5 °Brix [4] and 0.68 to 1.06% tartaric acid [23].

‘Bordô’ and ‘BRS Violeta’ grape juices grown using a low trellis system exhibited higher pH and lower titratable acidity (Table 1). Higher levels of reducing sugars were found in ‘Isabel’ grape juices, irrespective of the training system used, which had a significant influence on sensory analysis (Table 1). Despite the lack of color, ‘Isabel’ grape juices were rated the highest by evaluators in terms of taste, reflecting the preference of Brazilian consumers for sweeter juices [24]. The lack of color in ‘Isabel’ grape juices can be mitigated by blending with other varieties during production, such as ‘BRS Violeta’, ‘BRS Carmem’, ‘BRS Rúbea’, ‘BRS Cora’, and ‘BRS Magna’ [25].

‘Bordô’ and ‘BRS Violeta’ grape juices showed a similar violet coloration when analyzed by spectrophotometric methods (620 nm), with values ranging from 3.13 to 3.22, with the highest value obtained using the high trellis system, which also had the highest levels of anthocyanins (Table 2 and Table 3), pigments that contribute to the color of beverages. The higher color values measured at 420, 520, and 620 nm contributed to the greater color intensity and hue of ‘Bordô’ and ‘BRS Violeta’ juices (Table 1).

Higher color intensity and hue were observed in ‘BRS Violeta’ juice when produced using the low trellis system, whereas the lowest values were found in ‘Isabel’ juices, regardless of the training system (Table 1). These results are consistent with the levels of anthocyanins, total phenolic compounds, and antioxidant activity (Table 2), as ‘BRS Violeta’ juices showed higher total levels of these compounds compared to ‘Isabel’ juices. A positive correlation was found between anthocyanin content, total phenolic compounds, and antioxidant activity, leading to the conclusion that darker-colored juices exhibit higher antioxidant activity, likely due to the presence of polyphenols such as anthocyanins [8,9]. The color intensity and hue values obtained in all juices can be considered high, indicating good coloration, comparable to commercial Brazilian juices where color intensity ranges from 5.37 to 21.12 and hue from 0.57 to 1.04 [4].

Color parameters obtained via colorimetry also showed that ‘BRS Violeta’ juices had a darker color, evidenced by lower values of brightness and chroma and a higher hue angle, where brightness varies on a scale from 0 to 100, with 0 being black (dark) and 100 being white (light), so the lower the value, the darker the juice. The opposite effect was observed for ‘Isabel’ grape juices, which showed higher values of brightness and chroma and a lower hue angle (Table 2). When analyzing the training systems, it was found that grape juices produced using high trellis systems had lower values of brightness and chroma and a higher hue angle, indicating juices with more intense colors and greater vividness (Table 2).

The ‘Bordô’ and ‘IAC 138-22 Máximo’ grape juices were considered to have intermediate coloration compared to ‘BRS Violeta’ and ‘Isabel’ juices, as they showed average values of brightness, chroma, and hue (Table 2). Thus, besides ‘BRS Violeta’, which is highly suitable for producing intensely colored and antioxidant-rich red wines and grape juices [8], ‘Bordô’ and ‘IAC 138-22 Máximo’ also demonstrate excellent potential to contribute to the color of juices and wines from other varieties. Higher levels of total monomeric anthocyanins were obtained in ‘BRS Violeta’ juices, with 84.15% more than in ‘Isabel’ juices (Table 2). Regarding the training systems, a greater accumulation of this pigment was observed in high trellis systems, with an 8.44% higher concentration than in low trellis systems (Table 2). Therefore, the use of high trellis in grape cultivation for juice and wine production enhances the interplay between developmental and environmental signaling pathways, promoting anthocyanin biosynthesis and influencing the synthesis of other flavonoids, thus affecting the composition and quality of the beverages.

Anthocyanins are natural pigments responsible for the blue, purple, or red colors of juices, playing a fundamental role in the functional quality and color expression of Vitis spp. derivatives. These pigments also directly influenced the parameters of brightness, chroma, color intensity, and hue of the juices (Table 1 and Table 2). Juices made with ‘BRS Violeta’ grapes showed higher values of anthocyanins, hue, color intensity, chroma, and lower brightness, opposite to what was observed in ‘Isabel’ juices. Therefore, it can be suggested that ‘BRS Violeta’ grapes are an important source of anthocyanins, producing intensely colored grape juices rich in anthocyanins and antioxidant activity.

The total monomeric anthocyanin content of the juices obtained with both training systems, except for ‘Isabel’ juices, was higher than those reported in the literature for Brazilian whole juices from *V. labrusca* and hybrids, ranging from 15.4 to 617.0 mg L^−1^ [26,27], and from commercial juices from Spain (129 to 535 mg L^−1^) [28]. This was also observed for total phenolic compounds, as the highest and lowest levels were obtained in ‘BRS Violeta’ and ‘Isabel’ juices, respectively. When analyzing the training system, it was found that using a high trellis system promoted higher accumulations (Table 2). The total phenolic compound content of the juices from all varieties and training systems aligns with values found in the literature for *V. labrusca* and hybrid grape juices produced in different regions of Brazil, ranging from 779 to 2647 mg L^−1^ [26,27]. However, in this study, we found that the total phenolic content of ‘IAC 138-22 Máximo’ and ‘BRS Violeta’ juices exceeded the maximum value reported by these authors, characterizing them as hybrids with potential sources of phenolic compounds for consumers. The importance of phenolic composition in grape juices is mainly related to its contribution to the sensory quality of the juice in terms of color, flavor, and antioxidant activity [1], and this was observed in this study, as these juices showed a darker color and higher antioxidant activity (Table 1 and Table 2).

The antioxidant activity of the juices ranged from 9.46 to 55.06 mM Fe L^−1^, 55.36 to 115.43 µg TEAC mL^−1^, and 48.94 to 95.48 µg TEAC mL^−1^ when determined via the FRAP, DPPH, and ABTS methods, respectively. The highest and lowest antioxidant activities were found in the juices of ‘BRS Violeta’ and ‘Isabel’ grapes, respectively (Table 1 and Table 2). Ranking the juices in order of higher antioxidant activities, regardless of the analysis method, they can be classified as ‘BRS Violeta’ > ‘Bordô’ > ‘IAC 138-22 Máximo’ > ‘Isabel’, again showing the strong influence of color on antioxidant activity, as this order was maintained in the colorimetric aspects of the juices (Table 1 and Table 2).

It is important to emphasize that the antioxidant activity of grape products, such as juices, is influenced not only by the quantity of phenolic compounds but also by their composition. Additionally, it is crucial to consider that other components such as carotenoids (lycopene, β-carotene), tocopherols, and ascorbic acid are involved in the antioxidant activity of these beverages and can act synergistically with phenolics [29].

### 3.2. Profile of Phenolic Compounds and Antioxidant Activity of Whole Grape Juices

For all individual phenolic compounds quantified by means of ultra-high performance liquid chromatography, there was a significant interaction between the training systems and scion varieties. Among the quantified anthocyanins, malvidin-3,5-diglucoside was the major compound, with concentrations ranging from 5.13 to 80.89 mg L^−1^, found in ‘Isabel’ grape juices from low trellis and ‘Bordô’ grape juices from high trellis, respectively (Table 3). This can be attributed to the fact that malvidin has a stronger, more purple appearance, characteristic of ‘Bordô’ juices. On the other hand, the least representative anthocyanin, i.e., found in lower concentrations, was cyanidin-3-O-glucoside, with its maximum concentration (2.22 mg L^−1^) obtained in ‘Isabel’ grape juices from high trellis (Table 3). This can be explained by the fact that cyanidin has a less intense, more pinkish color, characteristic of ‘Isabel’ juices, and also because this genotype has low levels of anthocyanins and color in the juices. However, the concentrations found in this study were higher than those reported by [3,30] in juices of the same genotype.

Although ‘Isabel’ grape is a *V. labrusca* species, where diglycosylated anthocyanins are predominant [31], the main anthocyanin found in the juices of this genotype in this study was malvidin-3-*O*-glucoside, with an average of 6.20 to 6.33 mg L^−1^ (Table 3). These data corroborate results obtained in previous studies with juices and wines made from the same grape genotype, in which the concentration of malvidin-3-*O*-glucoside was higher than that of malvidin-3,5-diglucoside [32,33]. However, when analyzing ‘Bordô’ juices, another *V. labrusca* genotype, we found that the predominant anthocyanin was a diglycosylated one (malvidin-3,5-diglucoside), leading us to believe that this characteristic is intrinsic to each genotype, i.e., related to genetic inheritance.

Regardless of the scion varieties and training systems, considering all quantified anthocyanins in this study, in descending order, we have malvidin-3,5-diglucoside > cyanidin-3,5-diglucoside > delphinidin-3-*O*-glucoside > malvidin-3-*O*-glucoside > peonidin-3-*O*-glucoside > cyanidin-3-*O*-glucoside, meaning that diglycosylated ones stood out with higher concentrations, as these are predominant in *V. labrusca* and hybrid grapes [31]. Among the quantified glycosylated anthocyanins, delphinidin-3-*O*-glucoside was the most representative, with the highest concentration occurring in ‘BRS Violeta’ juices grown on high trellis. These results demonstrate that this training system is capable of influencing biosynthesis and promoting the accumulation of compounds that not only enhance the color of beverages but also provide health benefits, as delphinidin acts as a chemotherapeutic agent to prevent the development and progression of cancer cells [23].

In addition to being the main compounds found in red wines and grape juices and playing a fundamental role in the sensory and functional quality of these beverages [34,35], anthocyanins belong to the class of flavonoids and are rapidly absorbed, possessing various beneficial functions. It is important to note that both the profile and content of polyphenols can vary due to several factors, including the cultivation system, fertilization practices, climate conditions, harvest timing, and the presence of pathogens. These influences, which encompass both biotic and abiotic factors, can significantly affect polyphenol composition. Therefore, training systems that promote greater biosynthesis and accumulation of these compounds in juices are of utmost importance. For instance, malvidin-3-*O*-glucoside, the main anthocyanin found in grapes, has anti-inflammatory properties, helps to balance and inhibit pro-inflammatory pathways, and benefits cardiovascular health [36]. Additionally, together with peonidin, it reduces the expression of inflammatory genes [37], while cyanidin-3-*O*-glucoside has beneficial activity against various chronic inflammatory diseases, improving insulin resistance through the activation of cellular antioxidant and cytoprotective genes [38]. Thus, the juices produced in this study are excellent sources of health-promoting compounds capable of minimizing or even controlling certain diseases, as they contain these phytochemicals in their composition.

Regardless of the training system, hybrid grape juices showed the highest concentrations of rutin, with the highest found in ‘IAC 138-22 Máximo’ juices from high trellis (5.35 mg L^−1^) (Table 3). It is important to note that sunlight influences the biosynthesis involved in the production of flavonols in plant tissues, and grapes highly exposed to daylight experience a significant increase in flavonol biosynthesis [39]. High trellis thus provided greater biosynthesis of this important compound, a flavonoid studied for its biological effects and potential antioxidants [40].

High trellis also elevated the concentrations of catechin, another flavonol identified in grape juices, with the highest concentration occurring in ‘BRS Violeta’ grape juices from high trellis, with levels 80.72% higher than ‘Bordô’ juices in the same training system (Table 3). Rutin and catechin, important and major flavonols in hybrid grape juices and *V. labrusca*, have high antioxidant activity [41], providing our juice beverages with potential antioxidant activity.

Even in low concentrations, trans-resveratrol was quantified in all juices, with concentrations higher than those in ‘Isabel’ juices (Table 3). This result explains a possible relationship between this bioactive compound and color, as ‘Isabel’ grapes have lower coloration compared to the other varieties studied. The highest concentration of resveratrol is found in the grape skin, a result of ultraviolet radiation, with important action on phenolic metabolism. Therefore, the darker the grape or juice color, the higher the concentration of this important bioactive. It is important to highlight that the concentrations obtained in all juices are within those reported in the literature for Brazilian whole grape juices of hybrid and *V. labrusca* varieties, where concentrations ranged from 0.00 to 1.10 mg L^−1^ [19,23]. Trans-resveratrol is one of the most widely studied phenolics currently and has been associated with various beneficial effects on human health such as bactericidal and fungicidal activity, cardioprotective action, and anticancer activity, as well as favoring the reduction in blood pressure, suppressing lipid peroxidation, improving oxidative status, and reducing lipid levels [41]. Therefore, juices with high levels of this compound, as obtained in this study, can be a good source of resveratrol for consumers.

‘BRS Violeta’ juices showed the highest concentrations of phenolic acids, except for gallic acid, which was higher in ‘Bordô’ juices grown in a low trellis system, and trans-cinnamic acid, which was not quantified in any juice (Table 3). Among the quantified phenolic acids, caffeic and chlorogenic acids were the most abundant in all juices, representing important acids in the composition of the whole grape juices elaborated in this study, as they play a role in the healing process [42]. Additionally, chlorogenic acid also reduces leukocyte influx and modulates angiogenesis and metalloproteinase expression, thus accelerating tissue repair [43].

Considering all individually quantified phenolic compounds, regardless of the phenolic class they belong to, in descending order, we have ‘BRS Violeta’ juices > ‘Bordô’ > ‘IAC 138-22 Máximo’ > ‘Isabel’ (Table 3). A high trellis system provided greater synthesis and accumulation of phenolic acids in ‘IAC 138-22 Máximo’ and ‘Isabel’ juices, while no influence of the training system was observed for ‘Bordô’ and ‘BRS Violeta’ juices, indicating that the training system is highly linked to its interaction with the scion variety. ‘BRS Violeta’ juices in both training systems showed the highest concentrations of phenolics, making them important sources of bioactive compounds, as oxidative stress is involved in the genesis of various diseases. Moreover, the phenolic compounds mainly present in grapes and their derivatives, such as juices, act beneficially by delaying or even preventing this process and have a positive correlation with their action on oxidative stress [6,42]. Therefore, it is suggested that the juices produced in this study present intense antioxidant properties strongly related to the concentration of phenolic compounds.

### 3.3. Sensory Analysis

**Figure 1:**
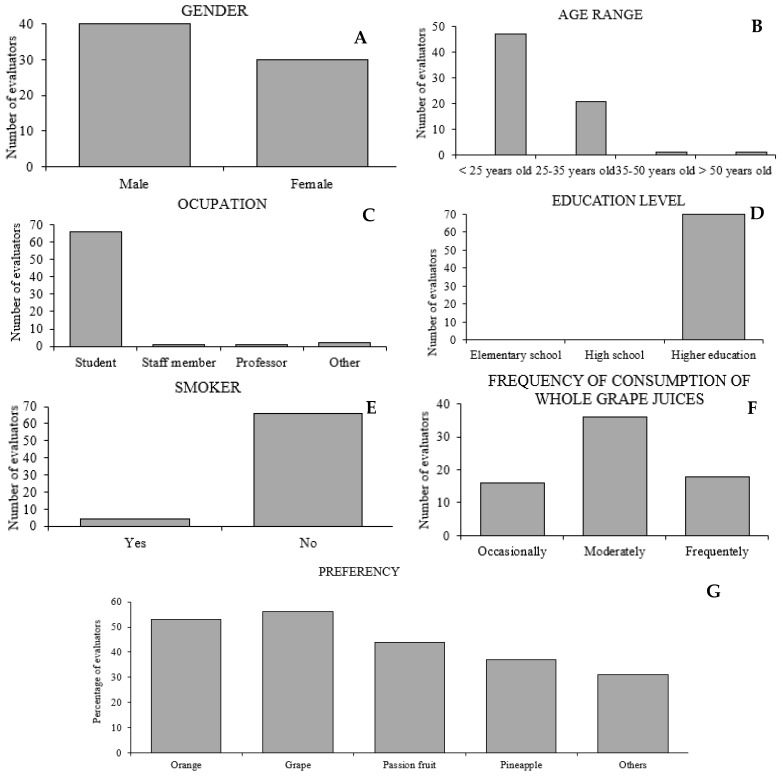
Sensory analysis. Gender (**A**), age range (**B**), occupation (**C**), education level (**D**), smokers (**E**), frequency of consumption of whole grape juices (**F**) and preferred fruit juice types (**G**).

The sensory profile of the seventy evaluators of whole grape juices was varied, with 57.14% being male and 42.86% female (Figure 1A). Regarding age group, occupation, and education, predominantly young individuals under 25 years old (67.14%) and students or graduates at the university level (94.29%) were represented (Figure 1B–D). The percentage of evaluators who consider themselves smokers was only 5.71% (Figure 1E); therefore, the results of this study were not affected by the evaluators’ palate, as tobacco affects this organoleptic characteristic. Regarding the frequency of consumption of whole grape juices, the majority of evaluators (51.43%) (Figure 1F) fell into the moderately frequent category (2–4 times a month), and concerning preferred fruit juice types, it was found that orange and grape juices were the favorites, representing 23.98% and 25.34%, respectively (Figure 1G). Thus, it is observed that grape juice is one of the preferred flavors among the evaluators, allowing us to infer that they are potential consumers of the juice under study. Overall, for the attributes of color, aroma, flavor, body, and overall acceptance, the juices from ‘Bordô’ and ‘Isabel’ grapes, both *V. labrusca* species, were the top-ranked, regardless of the training system (Table 4).

**Figure 2:**
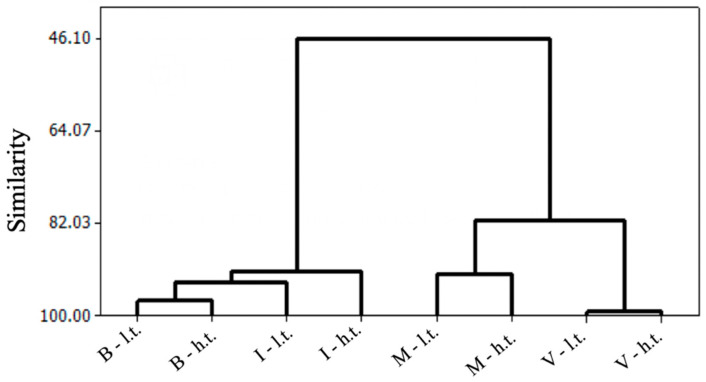
Intention to purchase whole juices from *Vitis labrusca* (‘Bordô’ and ‘Isabel’) and hybrid (‘IAC 138-22 Máximo’ and ‘BRS Violeta’) grapes in different training systems (low and high trellis). The similarity values on the y-axis represent the proximity of consumer responses regarding purchase preferences. The lower the similarity value, the greater the difference in preferences between the grape and training system options. Note: I-l.t. = ‘Isabel’ low trellis; I-h.t. = ‘Isabel’ high trellis; M-l.t. = ‘IAC 138-22 Máximo’ low trellis; M-h.t. = ‘IAC 138-22 Máximo’ high trellis; V-l.t. = ‘BRS Violeta’ low trellis; V-h.t. = ‘BRS Violeta’ high trellis; B-l.t. = ‘Bordô’ low trellis; B-h.t. = ‘Bordô’ high trellis.

Regarding the color of the juices, a characteristic related to the concentration of anthocyanins, all juices were ranked higher than ‘Isabel’ juices, as this variety has a deficiency in coloration, requiring blends with teinturier varieties, such as ‘BRS Violeta’, ‘BRS Rúbea’, ‘BRS Cora’, ‘BRS Carmem’, and ‘BRS Magna’ [25]. Coloration is an important quality component for grapes destined for processing, as in juice production, berry color significantly influences the final product. ‘Bordô’ grape juices from a high trellis system scored higher in the color attribute (Table 4), which was directly reflected in overall acceptance and purchase intention. It is noteworthy that these juices were classified as ‘definitely would buy’ by the evaluators (Figure 2), mainly due to their intense color, altering the visual quality for potential consumers (Table 4 and Figure 2). According to [44], grape juices with a more intense color have higher overall acceptance compared to those with a lower color intensity.

‘Bordon’ and ‘Isabel’ grape juices, regardless of the training system, were ranked as the most aromatic and flavorful (Table 4), a characteristic of *V. labrusca* species, which have a predominantly “foxy” aroma. Despite having high concentrations of anthocyanins and phenolic compounds (Table 2 and Table 3), responsible for the color, aroma, flavor, and body of the beverages, ‘BRS Violeta’ grape juices, regardless of the training system, were ranked the lowest in terms of flavor and body. This result is attributed to the presence of tannins, which directly contribute to the astringency and bitterness of the beverages. Thus, changes in the levels of these substances can greatly influence the flavor and body characteristics of the juices, which likely directly affected the acceptance of this grape variety’s juices.

‘Bordô’ grape juices, regardless of the training system, were the most intended to be “certainly purchased” by the evaluators (Figure 2), reflecting the high ranking obtained in the color characteristic of these juices (Table 1 and Table 2), as the color of this beverage is directly related to consumer acceptance. Even with a less intense color, ‘Isabel’ grape juices, regardless of the training system, were also indicated in the class of “certainly purchased” (Figure 2), reflecting the flavor of these juices, which had higher levels of soluble solids and sugars in their composition than the other evaluated juices (Table 1). The Brazilian consumer’s palate is accustomed to sweet beverages [11]. Therefore, despite having deficiencies in their coloration, ‘Isabel’ grape juices are well accepted in Brazil, as this variety is one of the most used for the production of Brazilian juices and wines [24].

### 3.4. Principal Component Analysis of Colorimetric, Physicochemical, and Biochemical Analyses

**Figure 3:**
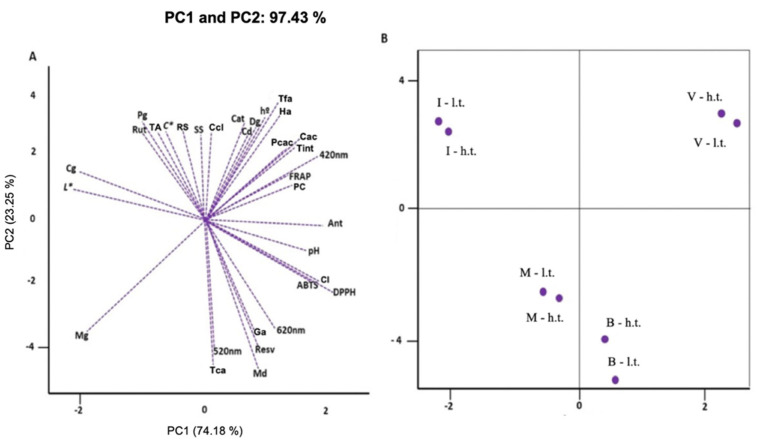
Principal components analysis of colorimetric, physicochemical and biochemical variables of whole grape juices for the interaction between two varieties of *Vitis labrusca* (‘Bordô’ and ‘Isabel’) and hybrids (‘IAC 138-22 Máximo’ and ‘BRS Violeta’) in different training systems (low and high trellis). Note: Soluble solids = SS; titratable acidity = TA; reducing sugars = RS; luminosity = L*; chroma = C*; hue angle = h°; yellow coloration = 420 nm; red coloration = 520 nm; violet coloration = 620 nm; color intensity = CI; hue = tint; anthocyanins = Ant; phenolic compounds = PC; antioxidant activity = DPPH, FRAP, ABTS; gallic acid = ga; 3-hydroxytyrosol acid = ha; catechin = cat; trans-cinnamic acid = tca; caffeic acid = cac; chlorogenic acid = ccl; *p*-coumaric acid = pcac; trans-ferulic acid = tfa; trans-resveratrol = resv; rutin = rut; cyanidin-3,5-dig = cd; delphinidin-3-*O*-g = dg; cyanidin-3-*O*-g = cg; malvidin-3,5-dig = md; peonidin-3-*O*-g = pg; malvidin-3-*O*-g = mg. (**A**) Plot of evaluated variables; (**B**) plot of combination insertion points. Note: I-l.t. = ‘Isabel’ low trellis; I-h.t. = ‘Isabel’ high trellis; M-l.t. = ‘IAC 138-22 Máximo’ low trellis; M-h.t. = ‘IAC 138-22 Máximo’ high trellis; V-l.t. = ‘BRS Violeta’ low trellis; V-h.t. = ‘BRS Violeta’ high trellis; B-l.t. = ‘Bordô’ low trellis; B-h.t. = ‘Bordô’ high trellis).

The interactions between the juices from different grape varieties and training systems were characterized by the first two principal components, which accounted for 97.43% of the variation between combinations (Figure 3).

The principal components efficiently grouped the different grape varieties from the training systems, showing that each variety has its own genetic characteristics, which will influence the quality attributes of the juices. For example, it was found that ‘Isabel’ grape juices, regardless of the training systems, had higher levels of reducing sugars, soluble solids, and luminosity and higher concentrations of the anthocyanins cyanidin-3-O-glucoside and peonidin-3-O-glucoside. However, these juices showed lower antioxidant activities (DPPH and ABTS) (Figure 3). Juices with less intense coloration have lower antioxidant activity than more colorful juices, as observed in ‘BRS Violeta’ juices, which had lower luminosity and a higher hue and color intensity, elevating the antioxidant activity of these juices (Figure 3).

‘BRS Violeta’ juices also had higher levels of anthocyanins and total phenolic compounds, and the concentrations of the anthocyanins cyanidin-3,5-diglucoside (cd) and delphinidin-3-*O*-glucoside (dg) and the phenolic acids caffeic, *p*-coumaric, 3-hydroxytyrosol, chlorogenic, and t-ferulic acids were also higher in these juices (Figure 3). The anthocyanins cyanidin-3-*O*-glucoside and peonidin-3-*O*-glucoside were found in higher concentrations in ‘Isabel’ juices, while in ‘BRS Violeta’ juices, higher levels of cyanidin-3,5-diglucoside (cd) and delphinidin-3-*O*-glucoside (dg) were observed. ‘Bordô’, ‘IAC 138-22 Máximo’, and ‘BRS Violeta’ showed the highest concentrations of trans-resveratrol. ‘Bordô’ juices also contained higher levels of gallic acid and malvidin-3,5-diglucoside, and ‘IAC 138-22 Máximo’ juices presented the anthocyanins malvidin-3,5-diglucoside and malvidin-3-*O*-glucoside, considered major components in these juices (Table 3 and Figure 3). Overall, the results obtained with the principal component analysis show that ‘BRS Violeta’ juices, regardless of the training system, had higher concentrations of bioactive compounds and antioxidant activity than the other juices (Table 2 and Table 3 and Figure 3).

## 4. Conclusions

The training system and grape variety are directly related to the presence of sugars, acids, and bioactive compounds in the composition of the juices. Juices made from ‘Bordô’, ‘IAC 138-22 Máximo’, and ‘BRS Violeta’ grapes stand out from ‘Isabel’ juices. Despite the low levels of anthocyanins and total phenolic compounds, ‘Isabel’ juices showed great acceptability by the evaluators, due to the high concentration of soluble solids and sugars, as Brazilian consumers prefer sweet beverages. ‘BRS Violeta’ juices have higher concentrations of bioactive compounds than the other juices. All juices made with different genotypes have considerable amounts of bioactive compounds, indicating juices with high antioxidant activity and, consequently, high biological potential. Additionally, the use of high trellises in vine cultivation promoted an increase in bioactive compounds, which could be an interesting way to improve the biological and physiological potential of whole grape juices.

## Figures and Tables

**Table 1:** Soluble solids, pH, titratable acidity, reducing sugars, coloration (420, 520, and 620 nm), color intensity, hue, and antioxidant activity (FRAP) in whole juices of *Vitis labrusca* grapes (‘Bordô’ and ‘Isabel’) and hybrids (‘IAC 138-22 Máximo’ and ‘BRS Violeta’) in different training systems.

Variables	Trellis	Varieties				CV (%)
		‘Bordô’	‘IAC 138-22 Máximo’	‘BRS Violeta’	‘Isabel’	
Soluble solids (°Brix)	Low	15.96 ± 0.15 bB	15.43 ± 0.15 bB	17.83 ± 0.06 aA	17.66 ± 0.06 aA	2.39
	High	16.76 ± 0.71 aAB	16.33 ± 0.81 aB	16.40 ± 0.36 bAB	17.30 ± 0.001 aA	
pH	Low	3.61 ± 0.08 aB	3.69 ± 0.07 aB	3.78 ± 0.01 aA	3.44 ± 0.01 aC	1.02
	High	3.51 ± 0.04 bB	3.62 ± 0.01 aA	3.63 ± 0.01 bA	3.46 ± 0.01 aB	
Titratable acidity (% tartaric acid)	Low	0.67 ± 0.07 bB	0.76 ± 0.06 aB	0.62 ± 0.11 bB	0.95 ± 0.06 aA	8.95
	High	0.84 ± 0.02 aAB	0.63 ± 0.06 bB	0.79 ± 0.04 aAB	0.99 ± 0.08 aA	
Reducing sugar (%)	Low	10.91 ± 0.08 aC	12.19 ± 1.06 bBC	13.97 ± 1.67 aAB	14.99 ± 1.41 aA	7.68
	High	12.24 ± 1.07 aB	14.07 ± 0.60 aAB	12.79 ± 1.08 aB	16.26 ± 0.04 aA	
420 nm (yellow)	Low	1.79 ± 0.42 bB	1.33 ± 0.08 bBC	3.50 ± 0.03 aA	0.99 ± 0.001 aC	13.36
	High	2.37 ± 0.61 aB	2.04 ± 0.22 aB	3.33 ± 0.08 aA	1.07 ± 0.02 aC	
520 nm (red)	Low	3.81 ± 0.04 aA	3.56 ± 0.02 bB	3.64 ± 0.001 aB	3.34 ± 0.01 bC	1.83
	High	3.81 ± 0.13 aA	3.91 ± 0.07 aA	3.45 ± 0.01 bB	3.60 ± 0.09 aB	
620 nm (violet)	Low	3.15 ± 0.07 aA	2.75 ± 0.14 bB	3.31 ± 0.12 aA	1.82 ± 0.07 aC	2.45
	High	3.22 ± 0.07 aA	3.00 ± 0.05 aB	3.13 ± 0.06 bAB	1.78 ± 0.04 aC	
Color intensity (CI)	Low	8.76 ± 0.46 bB	7.65 ± 0.21 bC	10.46 ± 0.14 aA	6.17 ± 0.08 aD	3.49
	High	9.40 ± 0.56 aAB	8.95 ± 0.10 aB	9.92 ± 0.12 bA	6.46 ± 0.11 aC	
Hue	Low	0.47 ± 0.10 bB	0.37 ± 0.02 bB	0.96 ± 0.01 aA	0.29 ± 0.001 aB	13.56
	High	0.62 ± 0.17 aB	0.52 ± 0.06 aB	0.96 ± 0.03 aA	0.29 ± 0.01 aC	
FRAP (mM Fe/L)	Low	27.73 ± 2.39 aB	14.66 ± 3.46 bC	55.06 ± 4.39 aA	9.46 ± 1.45 aC	9.67
	High	31.56 ± 1.55 aB	22.66 ± 3.66 aC	49.46 ± 1.84 bA	10.20 ± 0.52 aD	

Means followed by the same lowercase letter in the column and uppercase letter in the row do not differ from each other according to Tukey’s test at a 5% probability level.

**Table 2:** Coloration (lightness, chroma, and hue), monomeric anthocyanins, total phenolic compounds (PC), and antioxidant activity (DPPH and ABTS) in whole juices of *Vitis labrusca* grapes (‘Bordô’ and ‘Isabel’) and hybrids (‘IAC 138-22 Máximo’ and ‘BRS Violeta’) in different training systems.

Variables	Varieties				CV (%)
	‘Bordô’	‘IAC 138-22 Máximo’	‘BRS Violeta’	‘Isabel’	
Lightness (L*)	20.50 ± 0.30 ab	20.54 ± 0.15 ab	20.32 ± 0.05 b	20.76 ± 0.23 a	1.14
Chroma (C*)	1.10 ± 0.15 b	1.35 b ± 0.24	1.26 ± 0.03 b	2.13 ± 0.37 a	13.81
Hue	22.45 ± 8.98 b	20.94 ± 4.46 b	43.73 ± 4.51 a	18.80 ± 2.33 b	19.81
Anthocyanins (mg/L)	829.99 ± 63.25 b	893.13 ± 31.42 b	1411.08 ± 77.30 a	223.59 ± 22.73 c	8.09
Phen. comp. (mg/L)	1768.81 ± 59.89 c	2787.33 ± 35.67 b	3357.36 ± 35.31 a	1187.42 ± 38.57 d	3.29
DPPH (µg TEAC/mL)	103.58 ± 8.38 b	79.60 ± 5.81 c	115.43 ± 5.52 a	55.36 ± 1.39 d	6.88
ABTS (µg TEAC/mL)	85.23 ± 0.82 b	68.98 ± 1.30 c	95.48 ± 0.88 a	48.94 ± 1.63 d	0.91
**Variables**	**Trellis**				**CV (%)**
	**Low**		**High**		
Lightness (L*)	20.55 ± 0.19 a		20.52 ± 0.31 a		1.14
Chroma (C*)	1.56 ± 0.54 a		1.36 ± 0.36 b		13.81
Hue	24.31 ± 10.61 a		28.65 ± 12.44 a		19.81
Anthocyanins (mg/L)	805.44 ± 35.83 b		873.45 ± 35.55 a		8.09
Phen. comp. (mg/L)	2209.56 ± 47.85 b		2340.90 ± 47.13 a		3.29
DPPH (µg TEAC/mL)	87.67 ± 24.50 a		89.31 ± 18.71 a		6.88
ABTS (µg TEAC/mL)	75.56 ± 24.94 a		73.75 ± 18.07 a		0.91

Means followed by the same lowercase letter in the row, within the same factor, do not differ from each other according to Tukey’s test at a 5% probability level.

**Table 3:** Phenolic compounds (mg L^−1^) in whole juices of *Vitis labrusca* grapes (‘Bordô’ and ‘Isabel’) and hybrids (‘IAC 138-22 Máximo’ and ‘BRS Violeta’) in different training systems.

Compounds	Varieties	Trellis		CV (%)
Anthocyanins		Low	High	
Cyanidin-3,5-dig.	‘Bordô’	3.68 ± 0.06 bA	3.98 ± 0.01 bA	4.06
	‘IAC 138-22 Máximo’	1.95 ± 0.001 cA	2.05 ± 0.02 bA	
	‘BRS Violeta’	78.67 ± 2.03 aA	76.48 ± 1.02 aB	
	‘Isabel’	-	-	
Malvidin-3,5-dig.	‘Bordô’	79.97 ± 4.40 aA	80.89 ± 3.81 aA	4.10
	‘IAC 138-22 Máximo’	55.86 ± 1.67 bA	54.99 ± 0.81 bA	
	‘BRS Violeta’	60.36 ± 2.02 bA	57.95 ± 0.65 bA	
	‘Isabel’	5.13 ± 0.001 cA	6.05 ± 0.12 cA	
Delphinidin-3-*O*-g.	‘Bordô’	3.48 ± 0.001 bcA	4.07 ± 0.36 cA	8.05
	‘IAC 138-22 Máximo’	3.90 ± 0.07 bB	7.80 ± 0.29 bA	
	‘BRS Violeta’	21.40 ± 1.75 aA	19.76 ± 0.25 aB	
	‘Isabel’	2.28 ± 0.03 cA	3.19 ± 0.04 cA	
Cyanidin-3-*O*-g.	‘Bordô’	0.10 ± 0.04 bB	0.21 ± 0.03 cA	15.07
	‘IAC 138-22 Máximo’	0.09 ± 0.001 bB	0.34 ± 0.07 bA	
	‘BRS Violeta’	-	-	
	‘Isabel’	0.40 ± 0.001 aB	0.50 ± 0.01 aA	
Peonidin-3-*O*-g.	‘Bordô’	0.26 ± 0.001 cB	0.31 ± 0.001 cA	2.64
	‘IAC 138-22 Máximo’	0.52 ± 0.02 bA	0.51 ± 0.01 bA	
	‘BRS Violeta’	0.17 ± 0.01 dA	0.16 ± 0.001 dA	
	‘Isabel’	2.10 ± 0.001 aB	2.22 ± 0.005 aA	
Malvidin-3-*O*-g.	‘Bordô’	1.75 ± 0.04 cA	1.74 ± 0.12 cA	7.77
	‘IAC 138-22 Máximo’	8.78 ± 0.20 aB	12.77 ± 1.02 aA	
	‘BRS Violeta’	0.31 ± 0.01 dA	0.30 ± 0.01 dA	
	‘Isabel’	6.20 ± 0.02 bA	6.33 ± 0.02 bA	
Flavonol				
Rutin	‘Bordô’	1.80 ± 0.06 dB	2.95 ± 0.15 dA	4.25
	‘IAC 138-22 Máximo’	5.28 ± 0.14 aA	5.35 ± 0.03 aA	
	‘BRS Violeta’	3.65 ± 0.26 bA	3.45 ± 0.23 cA	
	‘Isabel’	2.52 ± 0.01 cB	4.57 ± 0.08 bA	
Estilbene				
*Trans*-resveratrol	‘Bordô’	0.05 ± 0.001 aA	0.05 ± 0.001 aA	0.37
	‘IAC 138-22 Máximo’	0.05 ± 0.001 aA	0.05 ± 0.001 aA	
	‘BRS Violeta’	0.05 ± 0.001 aA	0.05 ± 0.001 aA	
	‘Isabel’	0.04 ± 0.001 bA	0.04 ± 0.001 bA	
Phenolic acids				
Gallic acid	‘Bordô’	3.02 ± 0.06 aA	2.75 ± 0.08 aB	3.55
	‘IAC 138-22 Máximo’	1.40 ± 0.03 cA	1.36 ± 0.06 cA	
	‘BRS Violeta’	2.16 ± 0.04 bA	2.16 ± 0.10 bA	
	‘Isabel’	0.84 ± 0.02 dB	1.29 ± 0.05 cA	
3-hydroxytyrosol acid	‘Bordô’	0.14 ± 0.01 bB	0.21 ± 0.01 bA	5.38
	‘IAC 138-22 Máximo’	0.06 ± 0.01 cA	0.07± 0.01 cA	
	‘BRS Violeta’	0.45 ± 0.01 aA	0.39 ± 0.01 aB	
	‘Isabel’	0.09 ± 0.01 cA	0.08 ± 0.01 cA	
Caffeic acid	‘Bordô’	9.19 ± 0.23 bA	7.86 ± 0.21 bB	2.20
	‘IAC 138-22 Máximo’	7.02 ± 0.19 bA	6.28 ± 0.07 cB	
	‘BRS Violeta’	9.82 ± 0.28 aA	9.66 ± 0.03 aA	
	‘Isabel’	6.55 ± 0.13 cB	8.01 ± 0.15 bA	
Chlorogenic acid	‘Bordô’	10.13 ± 0.30 dA	9.54 ± 0.29 dB	2.59
	‘IAC 138-22 Máximo’	10.88 ± 0.36 cA	11.01 ± 0.20 cA	
	‘BRS Violeta’	14.31 ± 0.30 aA	14.17 ± 0.38 aA	
	‘Isabel’	13.15 ± 0.22 bA	13.06 ± 0.23 bA	
*p*-coumaric acid	‘Bordô’	0.28 ± 0.01 bA	0.24 ± 0.001 bB	6.74
	‘IAC 138-22 Máximo’	0.07 ± 0.001 cA	0.08 ± 0.001 cA	
	‘BRS Violeta’	0.46 ± 0.001 aB	0.52 ± 0.04 aA	
	‘Isabel’	0.07 ± 0.001 cB	0.10 ± 0.001 cA	
*trans*-cinnamic acid	‘Bordô’	-	-	-
	‘IAC 138-22 Máximo’	-	-	
	‘BRS Violeta’	-	-	
	‘Isabel’	-	-	
*trans*-ferulic acid	‘Bordô’	0.21 ± 0.01 bA	0.22 ± 0.02 bA	3.12
	‘IAC 138-22 Máximo’	0.15 ± 0.001 cA	0.15 ± 0.001 cA	
	‘BRS Violeta’	0.63 ± 0.001 aA	0.61 ± 0.001 aB	
	‘Isabel’	0.14 ± 0.001 cA	0.13 ± 0.001 dA	
Flavan-3-ol				
Catechin	‘Bordô’	4.57 ± 0.16 bA	4.21 ± 0.13 cA	3.65
	‘IAC 138-22 Máximo’	3.67 ± 0.11 cB	4.93 ± 0.03 cA	
	‘BRS Violeta’	20.76 ± 0.79 aB	21.84 ± 0.19 aA	
	‘Isabel’	2.12 ± 0.03 dB	8.68 ± 0.15 bA	
Total	‘Bordô’	118.78 ± 3.71 bA	119.40 ± 4.04 bA	2.80
	‘IAC 138-22 Máximo’	99.74 ± 2.82 cB	107.77 ± 0.34 cA	
	‘BRS Violeta’	213.24 ± 7.46 aA	207.55 ± 0.41 aA	
	‘Isabel’	41.67 ± 0.36 dB	54.29 ± 0.91 dA	

Means followed by the same lowercase letter in the column and uppercase letter in the row do not differ from each other according to Tukey’s test at a 5% probability level.

**Table 4:** Ranking of sensory analysis (hedonic scale) of whole juices of *Vitis labrusca* grapes (‘Bordô’ and ‘Isabel’) and hybrids (‘IAC 138-22 Máximo’ and ‘BRS Violeta’) in different training systems (low and high trellis).

Whole Grape Juices	Color	Aroma	Flavor	Texture	Overall Acceptance
‘Bordô’ low	296.21 ab	361.28 a	328.06 ab	312.05 a	337.22 a
‘Bordô’ high	309.47 a	356.16 a	335.74 ab	325.16 a	336.64 a
‘IAC 138-22 Máximo’ low	305.17 ab	228.47 b	282.21 bc	293.04 a	272.92 b
‘IAC 138-22 Máximo’ high	305.79 ab	213.65 b	258.34 c	279.28 a	248.51 b
‘BRS Violeta’ low	266.19 abc	209.46 b	174.84 d	215.21 b	176.11 c
‘BRS Violeta’ high	270.00 abc	220.90 b	174.64 d	217.14 b	189.54 c
‘Isabel’ low	253.19 bc	325.49 a	369.85 a	307.75 a	349.06 a
‘Isabel’ high	237.97 c	328.59 a	320.33 ab	294.36 a	334.00 a

Means followed by the same lowercase letter in the column do not differ from each other according to the Kruskal–Wallis test at a 5% probability level.

## Data Availability

Data is contained within the article.
